# Detection of Sulfonamide Antibiotics Using an Elastic Hydrogel Microparticles-Based Optical Biosensor

**DOI:** 10.1021/acsami.4c08010

**Published:** 2024-09-13

**Authors:** Veronika Riedl, Lara Heiser, Matthias Portius, Jann Ole Schmidt, Tilo Pompe

**Affiliations:** † Institute of Biochemistry, Leipzig University, Johannisallee 21-23, 04103, Leipzig, Germany

**Keywords:** Surface functionalization, Competitive binding, Hydrogel microparticles, Sulfonamide antibiotics, Environmental monitoring, Optical biosensor

## Abstract

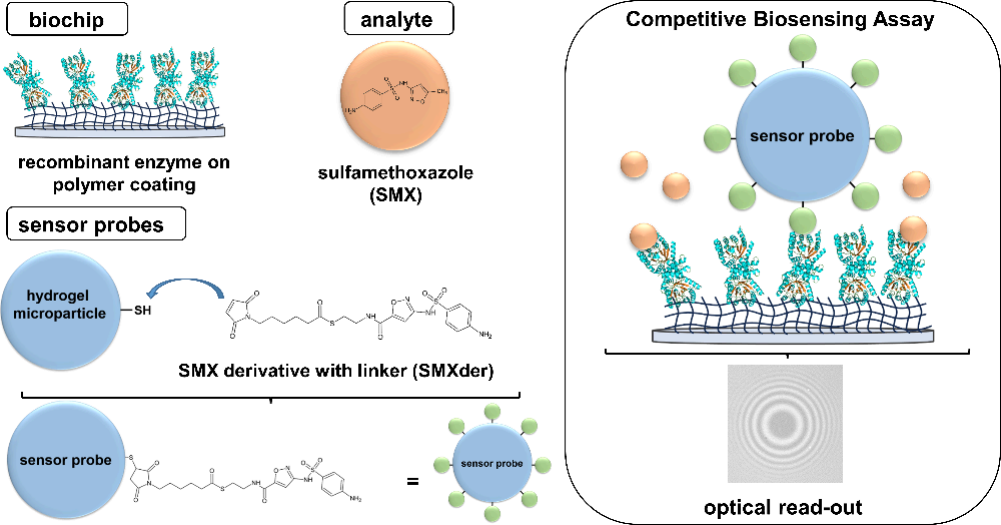

Sulfonamide antibiotics were the first synthetic antibiotics on the market and still have a broad field of application. Their extensive usage, wrong disposal, and limited degradation technologies in wastewater treatment plants lead to high concentrations in the environment, resulting in a negative impact on ecosystems and an acceleration of antibiotic resistance. Although lab-based analytical methods allow for sulfonamide detection, comprehensive monitoring is hampered by the nonavailability of on-site, inexpensive sensing technologies. In this work, we exploit functionalized elastic hydrogel microparticles and their ability to easily deform upon specific binding with enzyme-coated surfaces to establish the groundwork of a biosensing assay for the fast and straightforward detection of sulfonamide antibiotics. The detection assay is based on sulfamethoxazole-functionalized hydrogel microparticles as sensor probes and the biomimetic interaction of sulfonamide analytes with their natural target enzyme, dihydropteroate synthase (DHPS). DHPS from *S. pneumoniae* was recombinantly produced by *E. coli* and covalently coupled on a glass biochip using a reactive maleic anhydride copolymer coating. Monodisperse poly(ethylene glycol) hydrogel microparticles of 50 μm in diameter were synthesized within a microfluidic setup, followed by the oriented coupling of a sulfamethoxazole derivative to the microparticle surface. In proof-of-concept experiments, sulfamethoxazole, as the most used sulfonamide antibiotic in medical applications, was demonstrated to be specifically detectable above a concentration of 10 μM. With its straightforward detection principle, this assay has the potential to be used for point-of-use monitoring of sulfonamide antibiotic contaminants in the environment.

## 1 Introduction

Antibiotics have been among the most used pharmaceuticals worldwide.^1^ With a broad application field in human and veterinary medicine, as well as in animal husbandry, their production and usage have been strongly increasing. Due to the widespread application and wrong disposal, ultimately, antibiotics and their residues end up in sewage systems. Even with wastewater treatment plants (WWTPs) in developed countries, there is a constant release into the environment, as many low molecular weight molecules such as antibiotics cannot be removed by the state-of-art WWTP technology.^2,3^ The subsequent antibiotics accumulation in the environment poses a great threat to human health by accelerating the development of antibiotic resistances and harming the ecosystems.^4,5^

Being the first synthetic antibiotics on the market, sulfonamides are still widely used, commonly in combinatory drugs, such as the combination of sulfamethoxazole (SMX) and trimethoprim.^6,7^ As a consequence, sulfonamide antibiotics are ubiquitously detected in wastewater, surface water, and the environment in high concentrations.^3,8^

Therefore, monitoring the distribution of antibiotics in the environment has become a crucial task all over the world. Certified laboratories offer the analysis of environmental samples with established techniques, mostly chromatography-based methods such as high-pressure liquid chromatography or spectroscopic methods.^9,10^ These methods are very sensitive in detecting low-molecular-weight molecules with high precision. However, the sample preparation and device-based analysis are lab-bound and cost- and time-intensive, requiring trained personnel to be operated. Also, they do not allow for point-of-use monitoring. In order to solve this problem, alternative cost-effective methods for point-of-use applications in the detection of SMX and other antibiotics are continuously developed, ranging from colorimetry over immunochemistry and electrochemistry to particle-based methods.^11−15^ However, they usually fail to provide sensitivity, specificity, low-budget analytical readouts, and stable assay preparation techniques.

As a promising alternative in this field, we recently introduced a new sensing platform based on a biomimetic recognition principle mediated through the interaction between functionalized elastic poly(ethylene glycol) hydrogel microparticles, so-called soft colloidal probes (SCP), and a biological recognition element immobilized on a glass chip surface. The platform was successfully applied for biosensing applications in a competition assay format.^16−19^ Therein, the analyte of interest in the sample solution competes for binding sites of the recognition element at the chip surface with the competitor molecules coupled to the SCP. This biomimetic competitive binding of free analyte and functionalized SCP controls the microparticle binding and deformation at the chip surface, which is readout by a straightforward microscopy-based optical approach. This assay allowed for the highly sensitive detection of the controversially discussed broadband herbicide glyphosate in the picomolar range, meeting the legal threshold for pesticides in German tap water.^16^ The biosensing assay was shown to also be applicable for the detection of estrogenic steroid compounds.^20^ However, as demonstrated in these previous works, the extension of the SCP assay to other anthropogenic targets depends on a detailed understanding and analysis of coupling the biological recognition element on the biochip surface as well as the coupling of the analyte competitor on the SCP surface. Furthermore, in order to meet the demands of on easy and broadly applicable assay to monitor sulfonamide antibiotics at point of use in the environment, robust coupling schemes for the biochip have to be designed and readout options have to be simple, cheap, and reliable. Previously used biological recognition element coupling schemes via hydrophobin fusion proteins or Ni-NTA His-tag coupling did not provide these features, while polydisperse SCPs from emulsion polymerization resulted in rather elaborate readout algorithms.^16,17,20,21^

Based on our previous work, we now aimed to develop a new biomimetic interaction principle for this assay to detect sulfonamide antibiotics. Following this concept, we recently developed the synthesis of a SMX derivative (SMXder) for the oriented coupling to SCPs.^22^ We now set off to explore the usage of DHPS as the biological recognition element, including its synthesis, functional coupling on chip surfaces, and proof of binding to functionalized SCPs in a competitive assay. As shown in the following, we demonstrate the detection of sulfonamide antibiotics in a micromolar range using the SCP assay principle.

## 2 Materials and Methods

### 2.1 Materials

The pET28b(+) expression vector with the *sulA* gene was purchased at Genscript (Rijswijk, Netherlands). Isopropyl-β-thiogalactoside (IPTG) was purchased from Glentham Life Sciences Ltd. (U.K.). Tris buffer, sodium chloride (NaCl), magnesium chloride (MgCl_2_), HEPES buffer, MOPS buffer, dimethyl sulfoxide (DMSO), imidazole, isopropanol, acetone, dithiothreitol (DTT), and acetic acid were purchased at Carl Roth GmbH and Co. KG (Germany). Sodium phosphate monobasic monohydrate, (3-aminopropyl)triethoxysilane, *N*-succinimidyl-6-maleinimido-caproate-linker, poly(ethylene-*alt*-maleic anhydride) (PEMA), sodium pyrophosphate decahydrate, hydrogen peroxide, Tween20, and sulfamethoxazole were purchased from Merck KGaA (U.S.). Ammonia and tetrahydrofuran were purchased from Grüssing GmbH (Germany). Primary His_6_-tag monoclonal antibody, fluorescein 5(6)-isothiocyanate, SuperSignal West Pico PLUS Chemiluminescent Substrate, and blocking buffer were obtained at Thermo Fisher Scientific (U.S.). Secondary horseradish peroxidase-coupled antibody was purchased at Cell Signaling Technology (U.S.). 5-(and-6)-Carboxytetramethylrhodamine succinimidyl ester (TAMRA SE) was purchased at Biotium (U.S.). DNase I and RNase A were obtained from Applichem (Germany). Protease inhibitor tablets were purchased from Roche (Switzerland). Poly(octadecene-*alt*-maleic anhydride) (POMA) was purchased from Alfa Chemistry (U.S.). Nickel resin was obtained from Takara Bio USA, Inc. PEG-thiol and PEG-maleimide (both 4-arm, 2 kDa) were purchased from Creative PEGworks (U.S.).

Water used was purified by a Milli-Q water purification system.

### 2.2 Production and Purification of DHPS Enzyme

Dihydropteroate synthase (DHPS) from *Streptococcus pneumoniae* was recombinantly produced using T7 Express Competent *E. coli* cells. Synthesis of the nucleic acid sequence of *sulA* gene encoding DHPS and the subsequent cloning into pET28b(+) expression vector was performed by GenScript (Rijswijk, Netherlands). Amino acid and nucleic acid sequences of generated DHPS fusion protein with an N-terminal His_6_-tag are listed in the Supporting Information, sections 2 and 3. T7 Express Competent *E. coli* cells were transformed with pET28b(+)_sulA (GenScript, Rijswijk, Netherlands) via heat shock.

For the recombinant protein production, main cultures were incubated at 30 °C on a shaker (120 rpm) until protein production was induced at an optical density of 0.4–0.5 by adding 1 mM IPTG (Glentham Life Sciences Ltd., UK) and proceeded overnight at 30 °C. The next day, cells were harvested by centrifugation at 4,200 g and 4 °C for 20 min. Supernatant was removed, and the resulting pellets were stored at −20 °C overnight. The following day, pellets were resuspended in resuspension buffer (100 mM Tris/HCl, 50 mM NaCl, 1 mM DTT, pH 7.5) that was prepared following previous reports with the modification of not using phenylmethylsulfonyl fluoride (PMSF) and ethylenediaminetetraacetic acid (EDTA).^23^ During resuspension, DNase I (Applichem, Germany) and RNase A (Applichem, Germany) were added together with 5 mM MgCl_2_ and protease inhibitor tablets (Roche, Switzerland). Then, the cells were mechanically disrupted via ultrasound treatment. Cell debris was separated from the supernatant (lysate) by centrifugation at 20,000*g* at 4 °C for 35 min. Then, 20 mM imidazole was added to the lysate, and DHPS was purified via immobilized metal ion affinity chromatography (IMAC) on nickel resin (Takara Bio USA, Inc., U.S.). IMAC purification was performed according to the protocol of the manufacturer (Takara Bio USA, Inc., U.S.A.) with additional performing more extensive column washing. Shortly, the IMAC-column was washed with water, followed by equilibration buffer (50 mM sodium phosphate, 300 mM sodium chloride, 20 mM imidazole, pH 7.4). Then, a lysate containing DHPS was applied twice. The column was washed with 10 CV equilibration buffer, followed by 15 CV washing buffer (50 mM sodium phosphate, 300 mM sodium chloride, and 40 mM imidazole, pH 7.4). DHPS was eluted by applying elution buffer (50 mM sodium phosphate, 300 mM sodium chloride, 300 mM imidazole, pH 7.4) to the column and collecting 1 mL fractions.

To remove imidazole and transfer DHPS into storage buffer (50 mM sodium phosphate monobasic monohydrate, 300 mM sodium chloride, pH 7.4) that was prepared following published protocols, with the difference of using a higher sodium chloride concentration (instead of 150 mM) as well as a higher pH (instead of pH 7.0).^23^ Size exclusion chromatography (SEC) was performed using the KTA purifier system (GE HealthCare, U.S.) with a Superdex 200 Increase 10/300 GL column (Cytiva, U.S.).

### 2.3 Biochemical Characterization of DHPS

To investigate DHPS in SDS-PAGE, samples were prepared with 2× loading dye followed by a 5 min incubation at 95 °C. SDS gels consisted of 4% stacking and 12% resolving gel. SDS-PAGE was run at 120 V for 2 h at room temperature (rt), and gels were subsequently stained with colloidal Coomassie brilliant blue for detection. For Western blot analysis, proteins were transferred to a PVDF membrane (Thermo Fisher Scientific, U.S.) performing semidry blotting at 160 mA and 4 °C for 80 min. Then, the membrane was blocked for 1 h at r.t. and incubated overnight at 4 °C with primary His_6_-tag monoclonal antibody (Thermo Fisher Scientific, U.S.), that was prepared as a 1:1,000 solution in 1× Tris-buffered saline with Tween20 (TBS-T). The next day, the membrane was washed with TBS-T, followed by an one h incubation with the secondary HRP-coupled antibody (Cell Signaling Technology, U.S.) at r.t.. Western Blot was performed using HRP coupled to the secondary antibody and SuperSignal West Pico PLUS Chemiluminescent Substrate (Thermo Fisher Scientific, U.S.). Additionally, the molecular weight of DHPS was confirmed via matrix-assisted laser desorption/ionization time-of-flight mass spectrometry (MALDI-ToF MS). MALDI-ToF MS measurements were performed at the Core Facility Mass Spectrometry at Leipzig University.

### 2.4 Fluorescent Labeling of DHPS

For fluorescent labeling of DHPS, TAMRA SE (Biotium, U.S.) was added at a 5× molar excess and incubated for 1 h in a rotary shaker at r.t. To remove excessive TAMRA SE dye afterward, SEC was performed using the ÄKTA purifier system with a HiTrap Desalting column (Cytiva, U.S.) and DHPS storage buffer, as eluent for the TAMRA-labeled DHPS (TAMRA-DHPS).

### 2.5 Surface Functionalization with Maleic Anhydride Copolymers and TAMRA-DHPS

Coverslips of 13 mm diameter (VWR, U.S.) were precleaned by ultrasonic treatment in water for 30 min, followed by 30 min in ethanol. All subsequent steps were performed according to established protocols in the lab with the modification of drying the coverslips before aminosilanization.^24,25^ Coverslips were RCA cleaned by heating a solution of hydrogen peroxide, ammonia, and water in a ratio of 1:5:5 to 65 °C and incubating the coverslips for 10 min at 65 °C. To introduce amino groups on the glass surface for further coupling reactions, coverslips were dried with nitrogen and incubated for 10 min in 20 mM (3-aminopropyl)triethoxysilane (Merck, U.S.) isopropanol/water solution (v/v ratio 100:1). In the next step, coverslips were washed in isopropanol three times, dried with nitrogen, and tempered for 1 h at 120 °C. For the polymer coating, PEMA was prepared as a 0.1% (w/v) solution in acetone and tetrahydrofuran at 1:2 ratio (v/v). The other maleic anhydride copolymer, POMA, was also prepared as a 0.1% (w/v) solution in tetrahydrofuran. Subsequently, polymers were applied to the coverslips with a POLOS Spin150i spin coater (SPS-Europe B.V., The Netherlands) for 30 s with 4,000 rpm using 40 μL of polymer solution for each surface. Subsequently, coated surfaces were tempered for 2 h at 120 °C. To remove polymer not covalently coupled to the surface, coverslips were placed in acetone for 15 min, washed three times in acetone, and dried with nitrogen. Before TAMRA-DHPS binding, the polymer surfaces were tempered at 120 °C for 2 h. Then, coverslips were covered with 300 μL of either a 0.4 or 4 μM TAMRA-DHPS solution and incubated for 1 h. Finally, coverslips were washed with water, dried with nitrogen, and transferred to a 24-well glass bottom plate (Greiner, Austria) for following microscopy experiments.

### 2.6 Confocal Laser Scanning Microscopy Analysis of DHPS Immobilization

Fluorescence microscopy was conducted using a confocal laser scanning microscope (Leica DMi8, Germany) with a 20×/0.55 HC PL FLUOTAR dry objective (Leica, Germany). Image z-stacks were acquired across the TAMRA-DHPS coated surface and analyzed using the image analysis software Fiji (ImageJ 1.53f51) to generate a maximum intensity image of the recorded image stack at each measured position on the coverslip surface.^26−28^ Median intensity values of all measured positions were averaged for each condition.

### 2.7 Synthesis of Hydrogel Microparticles

Microfluidic synthesis was performed following the work of Rettke et al. with the modification of resolving the PEG in acetic acid instead of in 1× phosphate buffered saline.^29^ Shortly, PEG-thiol and PEG-maleimide (both four-arm, 2 kDa) were dissolved in acetic acid (0.1 M, pH 4.0), followed by a 10 min treatment in an ultrasonic bath. After centrifugation at 15000*g* at 20 °C for 10 min, the supernatant was removed and used in the subsequent microfluidic synthesis. Synthesized hydrogel microparticles were washed following the protocol of Rettke et al.^29^ Synthesis resulted in hydrogel microparticles of approximately 50 μm in diameter.

### 2.8 Functionalization of Hydrogel Microparticles with a Sulfamethoxazole Derivative (SMXder)

Hydrogel microparticles were functionalized with a synthesized sulfamethoxazole derivative (SMXder) according to Riedl et al.^22^ A minor variation from the reported protocol was using a *N*-succinimidyl-6-maleinimido-caproate-linker, resolved in a HEPES buffer/5% DMSO solution, to couple SMXder to the particle surface.

### 2.9 Fluorescent Labeling and Microscopic Analysis of Functionalized Hydrogel Microparticles

Fluorescent labeling of hydrogel microparticles with FITC and microscopy analysis were performed in accordance to Riedl et al.^22^

### 2.10 SCP-Based Biosensing Assay

For the biosensing assay, 4 μM DHPS was bound to coverslips coated with maleic anhydride copolymers, as described in section 2.5. DHPS-covered coverslips were incubated for 15 min at r.t. with the reaction solution consisting of MOPS buffer (0.1 M, pH 6.0) with 5 mM magnesium chloride, 600 μM sodium pyrophosphate decahydrate and sulfamethoxazole in the concentrations 1 μM, 10 μM, 100 μM, and 1 mM. Finally, 25 μL of SCPs were added and incubated for 15 min.

### 2.11 Reflection Interference Contrast Microscopy

Hydrogel microparticle contact areas resulting from sedimentation and competitive binding to DHPS immobilized on the biochip surface were documented using an inverted Olympus IX73 microscope (Olympus, Germany) with a 60×/1.35 UPlanSApo objective (Olympus, Germany), a monochromatic 530 nm collimated LED (M530L2-C1, Thorlabs, Germany) and a 50/50 beam splitter (Olympus) in the incident beam, enabling reflection interference contrast microscopy. Images of contact areas were recorded from single particles with an exposure time of 100 ms using Micro-Manager 2.0.0 software.^30,31^ The diameter of each contact area was determined using Fiji (ImageJ 1.53f51).

### 2.12 Statistical Analysis

Evaluated data were plotted with GraphPad Prism version 8.0 for Windows (GraphPad Software, Dotmatics, California, US). Plotted data are presented as the mean value with standard deviation (SD), if not stated otherwise. Statistical analysis was performed using a nonparametric Kruskal–Wallis test with Dunn’s multiple comparison test. Differences were evaluated as significant for *P* < 0.001 (***), *P* < 0.01 (**), and *P* < 0.05 (*).

## 3 Experimental Setup and Operating Principle for the SCP-Based Detection of Sulfonamide Antibiotics

**Figure 1:**
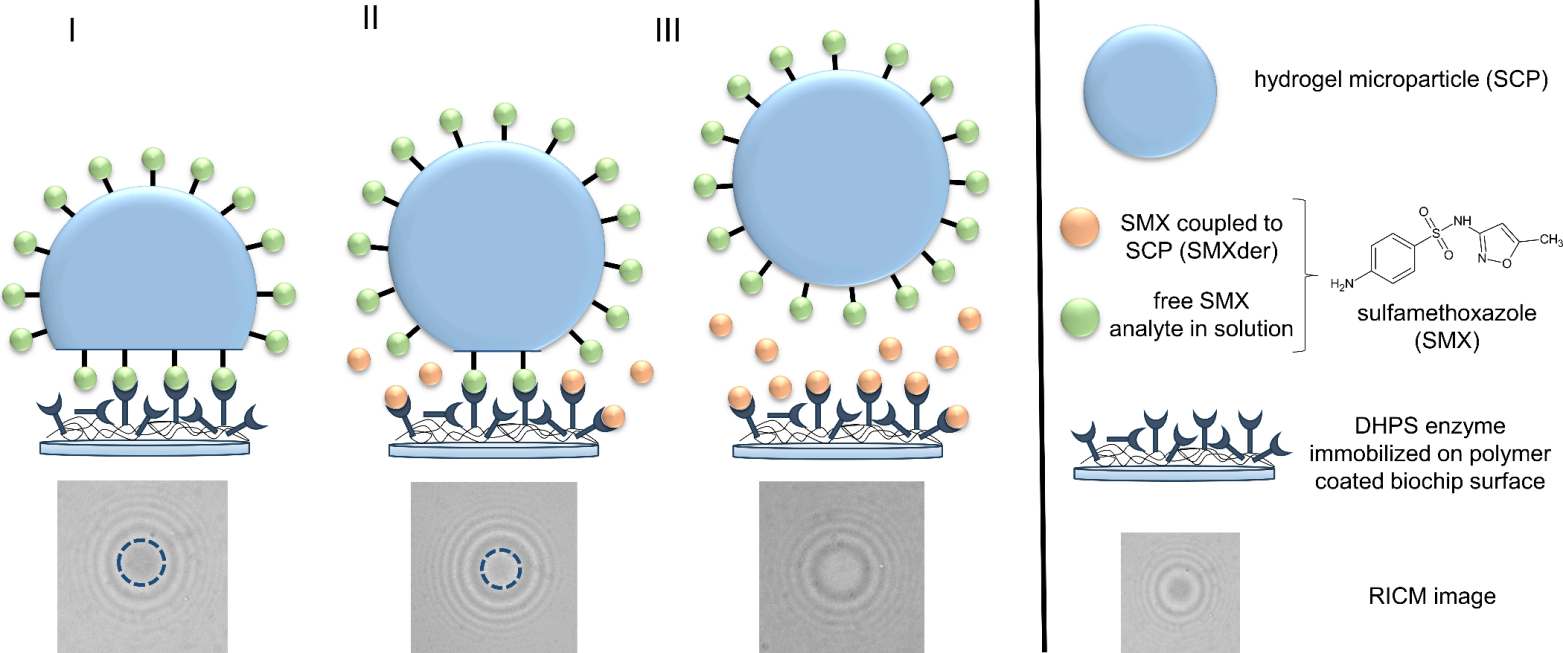
Overview of the experimental setup and general operating principle of the SCP-based biosensor for sulfonamide detection. All depicted components are described in the right-hand panel. Panels I–III show three possible states of the competitive biosensing SCP assay: (I) SCP functionalized with sulfamethoxazole (SMXder) applied to the biochip surface with no free SMX present in the sample solution. SMXder on the SCP binds to the immobilized DHPS enzyme on the biochip surface forming a large contact area due to elastic deformation of SCP. The contact area (central dark area of RICM interference pattern highlighted by a dashed blue line) is detected via reflection interference contrast microscopy (RICM). (II) A sample containing a low concentration of free SMX leads to a reduced binding of the SCP to the biochip surface due to competitive blocking of DHPS binding sites. Consequently, the size of the contact area is decreased in a concentration-dependent manner, which can be used to calibrate contact area to analyte concentration. (III) At a high SMX concentration in the sample solution, binding of SCP to the biochip surface and thus formation of a contact area is prevented.

To expand the SCP-based sensing platform for sulfonamide antibiotic detection, we used a specific biological recognition element, the sulfonamides’ natural target enzyme DHPS. Furthermore, we explored a new DHPS immobilization strategy and a new coupling strategy for SMXder to the SCP. Schemes of our experimental setup and the general SCP sensing principle are depicted in Figure 1.

To perform the competitive binding assay, an aqueous sample containing sulfonamide antibiotics is applied to the biochip, where sulfonamides are bound by biological recognition elements immobilized on the surface. We used SMX as a representative sulfonamide antibiotic due to its high usage in the medical field and ubiquitous presence in the environment. Thereupon added functionalized SCPs sediment and interact with the DHPS functionalized chip surface. Due to their low stiffness, SCPs undergo elastic deformation upon their specific binding to the DHPS via the surface-coupled SMXder. The resulting contact area can be optically detected via reflection interference contrast microscopy (RICM). Since sulfonamide analytes in the sample solution block DHPS binding pockets on the biochip surface in a concentration dependent manner, available binding sites for the SCP-biochip interaction also depend on the sulfonamide concentration. As the contact area is related to this SCP-biochip interaction, according to colloidal particle interaction theory,^32^ a calibration curve can be generated, allowing the determination of unknown sulfonamide concentrations in sample solutions.

## 4 Results and Discussion

### 4.1 DHPS Protein Production and Analysis

**Figure 2:**
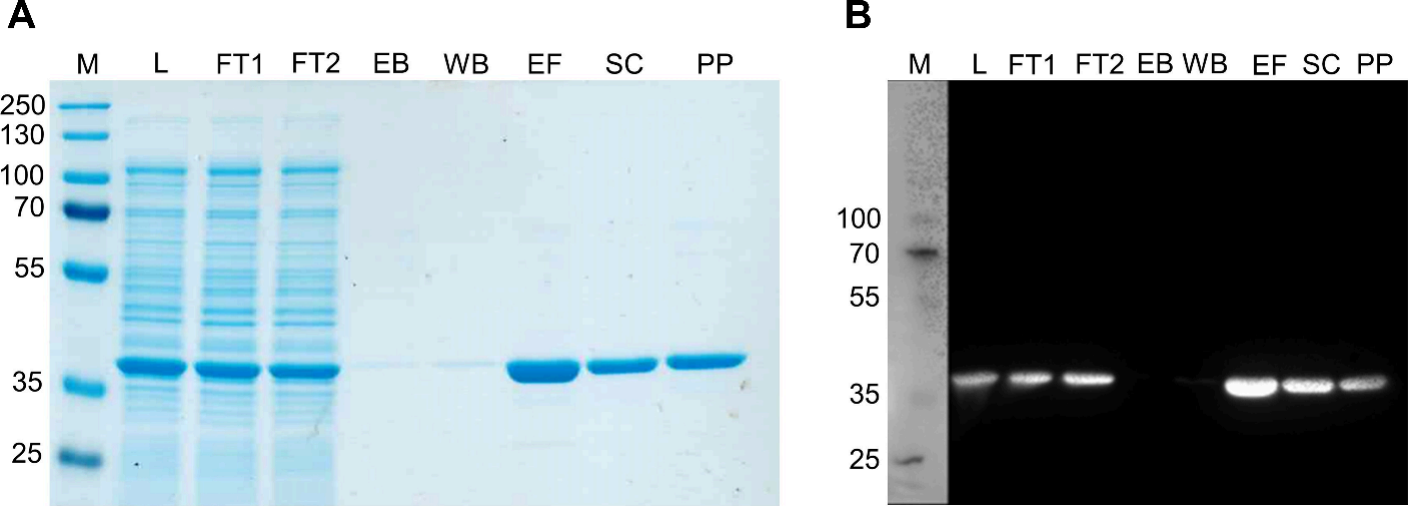
Purification and analysis of DHPS after recombinant production in *E. coli*. DHPS purification via IMAC and SEC confirmed by SDS-PAGE (A) and Western Blot analysis (B) on SDS gels with DHPS protein bands at an expected molecular weight of 36.9 kDa. Images A and B do not show the same gel; however, samples were prepared and applied to the gels analogously. Protein bands on the SDS-PAGE were detected by Coomassie colloidal staining. Protein on the Western Blot membrane was detected via the His-tag using chemiluminescence. The chemiluminescence image was superimposed with a standard protein ladder image of the same gel to visualize protein size in (B). Applied samples show the clear lysate after cell disruption (L), first and second flow through (FT1, FT2) after application of clear lysate on an IMAC column, washing steps of IMAC column with equilibration (EB) and washing (WB) buffer, elution of DHPS from the IMAC column (EF), DHPS directly after size exclusion chromatography (SC) and after concentration via centrifugal filter (purified protein, PP).

We chose the dihydropteroate synthase (DHPS) enzyme as recognition element for our detection setup as it is the natural target of sulfonamide antibiotics in bacteria and other microorganisms exhibiting a high and specific affinity to bind sulfonamides.^33^ The enzyme has a molecular weight of 34 kDa and fuels the folate biosynthesis pathway by catalyzing the condensation reaction between its two substrates *p*ABA and DHPPP to form 7,8-dihydropteroate.^34^ During this reaction, DHPPP is bound in one of the β-barrels of the DHPS dimer. Sulfonamide antibiotics competitively inhibit the binding of *p*ABA leading to the inhibition of bacterial growth.^35^ We performed recombinant DHPS production following reported approaches with some modifications that are listed in the experimental section.^36,37^ In a first step, the *sulA* gene encoding DHPS was synthesized and cloned into a pET28b (+) expression vector by GeneScript (Rijswijk, Netherlands). Then, recombinant protein production was performed in SHuffle T7 Express *E. coli* cells resulting in a fusion enzyme exhibiting an N-terminal His_6_-tag. Using this His-tag, DHPS was purified via IMAC followed by SEC as a second purification step. Successful production and purification of DHPS was confirmed by SDS-PAGE and Western Blot analysis showing constituent protein bands at the expected molecular weight (Figure 2) as well as size exclusion chromatography data showing a distinct absorbance signal at 280 nm during protein elution (Supporting Information, Figure S1B).

DHPS production resulted in a yield of 5.7 mg/L of the bacterial culture. DHPS molecular weight was determined to be 36.9 kDa from SDS-PAGE and Western Blot analysis as well as matrix-assisted laser desorption/ionization time-of-flight (MALDI-ToF) mass spectrometry (Supporting Information, Figure S1A). This result agrees with other biochemical characterizations of DHPS indicating a molecular weight of 34 kDa. The slight molecular weight increase of 2.9 kDa compared to the wild type protein is a result of the His-tag as well as restriction enzyme cutting sites fused to the N-terminus of the enzyme.^36,38^

### 4.2 Biochip Surface Modification and Coupling of DHPS as Recognition Element

**Figure 3:**
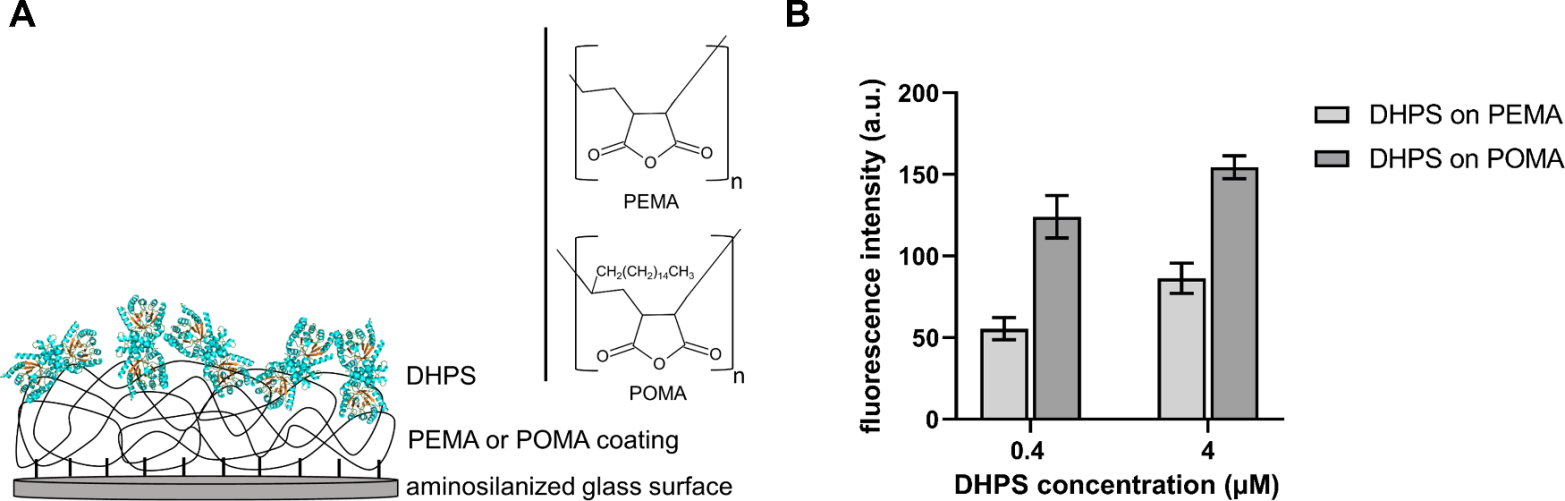
Biochip surface functionalization with DHPS as recognition element. (A) Scheme of DHPS immobilized on polymer-coated biochip surface showing nonoriented immobilization on either poly(ethylene-*alt*-maleic anhydride) (PEMA) or poly(octadecene-*alt*-maleic anhydride) (POMA) polymer. (B) Fluorescence intensity of TAMRA-labeled DHPS immobilized on either PEMA or POMA at two different solution concentrations (0.4 and 4 μM), analyzed via confocal laser scanning microscopy. Fluorescence intensity is shown as mean ± SD (2 samples with 15 measurements each).

In the next step, we investigated the feasibility of a direct DHPS coupling to the glass surfaces in a nonoriented manner to receive biochips with biomimetic recognition elements. We used a covalent coupling scheme for the formation of stable amide bonds via reactive anhydride copolymer coating on glass surfaces. Free amine groups of the protein react with anhydride groups for covalent immobilization, as previously shown.^25^ Thin layers of either PEMA or POMA were bound to the biochip surface by aminosilanization and subsequent spin-coating (Figure 3A). Copolymers such as PEMA or POMA have the advantage of being chemically versatile coatings with a high density of accessible reactive anhydride groups. Additionally, variation of parameters such as the polymer layer thickness by applying different concentrations of the polymer solution during spin-coating as well as surface hydrophobicity, with PEMA being hydrophilic and POMA being hydrophobic, allow for the regulation of protein density and conformation of coupled enzymes.^24,25^

To semiquantitively study the concentration-dependent immobilization of DHPS in the range of 0.4 to 4 μM, we used TAMRA-labeled DHPS and analyzed the fluorescence intensity of DHPS bound to the biochip surfaces using confocal laser scanning microscopy. For both polymer coatings an increase in fluorescence intensity was observed by increasing DHPS concentration from 0.4 to 4 μM (Figure 3B).

POMA surfaces with immobilized DHPS exhibited a higher fluorescence intensity for both DHPS concentrations compared to PEMA. Our findings are in accordance with previous works on protein immobilization, especially on maleic anhydride copolymers, reporting dense protein coverage at protein solution concentrations around 100 μg/mL (corresponding to 3 μM DHPS concentration) and higher amounts of immobilized proteins on hydrophobic surfaces.^24,39^ The increase in fluorescence intensity upon an increase in protein concentration shows a successful immobilization of DHPS at high surface density for both copolymer coatings on the biochip. For all further experiments we applied 4 μM DHPS to the biochip surface as the 10-fold increase in DHPS concentration only led to a minor increase in surface coverage, indicating almost full surface coverage, in accordance with previous studies.^24,39^

Enzyme immobilization on a biochip crucially affects the stability and sensitivity of a biosensor.^40−42^ Physical adsorption and covalent coupling with oriented and nonoriented immobilization are applied schemes.^43,44^ Aiming at developing a biosensor for point-of-use measurements in the environment, a stable and robust coupling scheme is absolutely essential. Based on previous successful enzyme immobilization approaches, we used a stable covalent coupling with maleic anhydride copolymers because of their chemical versatility and stability and the options to vary surface hydrophobicity, in contrast to other straightforward covalent coupling alternatives like glutaraldehyde frequently used for covalent enzyme immobilization in biosensing applications.^25,42,45,46^

Already in previous SCP assay studies, several covalent coupling schemes of recognition elements were applied, such as self-assembly of hydrophobin fusion proteins, as well as site-directed coupling through cooperative binding of His-tag proteins on Nickel (Ni)-NTA functionalized surfaces. Those site-directed methods can help to better define the enzyme orientation, improving the availability of the binding sites of the recognition element. However, they can also bear various disadvantages, such as laborious and low-yield protein production procedures for the highly hydrophobic hydrophobins, as well as the possibility of interference of fusion tags with substrate binding. Also, the low stability of Ni-NTA coupling in complex sample solutions, e.g., in the presence of metal ions or chelators such as ethylenediaminetetraacetic acid (EDTA) poses a challenge.^16,20,47^ Therefore, those biochip coupling schemes were omitted in the current study in order to explore the possibility of a simpler protein production and a straightforward nonoriented immobilization procedure with high stability.

### 4.3 Functionalization and Analysis of SCP with Sulfamethoxazole

For the functionalization of SCP with SMXder, we followed our recently reported approach of SMXder synthesis and subsequent coupling to the SCP surface.^22^ Shortly, the methyl group at the isoxazole ring of SMX was substituted with a thiol group in the SMXder synthesis that could be used for further coupling procedures. After microfluidic synthesis of SCP using 4-arm PEG precursors with thiol and maleimide groups for cross-linking by thiol–ene Michael addition reaction, SCPs bear mainly thiol groups on the surface due to the fast hydrolysis of maleimide groups.

**Figure 4:**
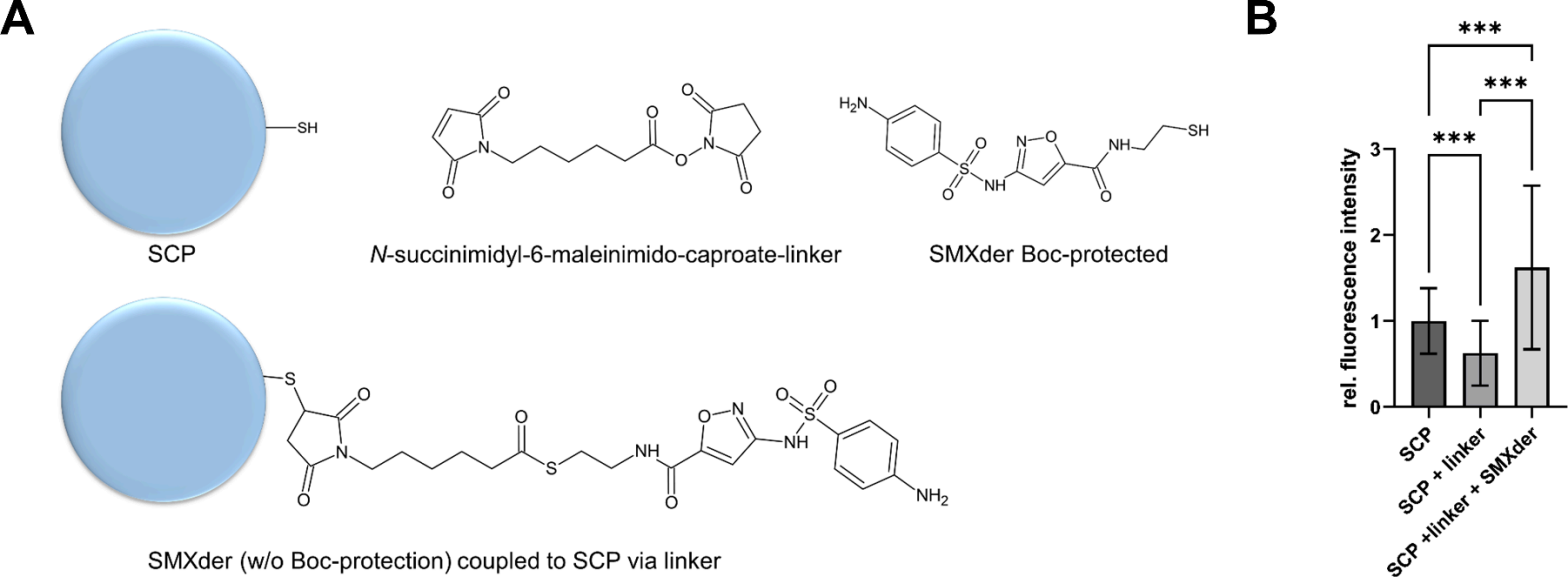
Sulfamethoxazole derivate (SMXder) coupling to SCP. (A) (Top) Overview depicting all components for SMXder coupling to SCP, that is SCP (unfunctionalized with exposed thiol groups), N-succinimidyl-6-maleinimido-caproate linker and SMXder with Boc protection group. (Bottom) Scheme of functionalized SCP with SMXder (w/o the Boc protection group) coupled via N-succinimidyl-6-maleinimido-caproate linker. (B) Fluorescence intensity analysis to verify SMXder coupling to SCP. Relative fluorescence intensity of SCP at various reaction steps are shown with nonfunctionalized SCP, N-succinimidyl-6-maleinimido-caproate linker coupled to SCP (SCP + linker) as well as SMXder coupled via linker to SCP (SCP + linker + SMXder). All SCP were masked with 10 mM *N*-methyl maleimide prior to FITC staining. Fluorescence intensity was normalized to nonfunctionalized SCP. Data are presented as mean ± SD of at least 390 SCP.

To couple the SMXder to the SCP, we targeted the thiol groups using a *N*-succinimidyl-6-maleinimido-caproate-linker as a spacer between SCP and SMXder (Figure 4A). In a two-step reaction, we coupled the bifunctional *N*-succinimidyl-6-maleinimido-caproate-linker via the maleimide group. Subsequently, the reactive ester of the linker was used to bind to SMXder in its thiol group. The more reactive amino group was kept nonavailable in the functionalization step by Boc protection.

After Boc-deprotection, we used confocal laser scanning microscopy, to investigate SMXder coupling to SCP by labeling the accessible amino groups with fluorescein 5(6)-isothiocyanate (FITC), as recently introduced in Riedl et al.^22^ To avoid FITC binding on the SCP surface generating unspecific signals, free thiol groups were masked with *N*-methyl maleimide prior to FITC staining. As shown in Figure 4B, coupling of the linker without SMXder to SCP resulted in a decrease in fluorescence intensity due to the linker occupying thiols that are still available after masking and that contributed to the background signal that was detected in the control condition. Coupling SMXder via the linker to SCP led to a significant increase in fluorescence intensity demonstrating the successful coupling of linker to SCP and the subsequent functionalization with SMXder.

### 4.4 Detection of Sulfonamides with the SCP Biosensing Assay

With the recombinantly produced DHPS immobilized on the polymer coated biochip surface and the SMXder coupled to SCP as sensor probes, we established all essential constituents to investigate the biomimetic interaction between the DHPS on the biochip surface and the SCP. To ensure the specific binding of functionalized SCP and free SMX analyte to DHPS, we had to consider that the DHPS exhibits a determined binding order of substrate and cosubstrate. Naturally, DHPS binds its first natural substrate DHPPP to induce the conformational change for binding the second substrate *p*ABA or sulfonamide antibiotics as its competitive structural analogues. However, DHPPP can be substituted by inorganic pyrophosphate (PP_i_) to induce the binding of *p*ABA or sulfonamide substrates without performing the subsequent catalytic reaction.^23^ Therefore, our biosensing assay buffer was chosen to contain PP_i_ as DHPPP surrogate as well as Mg^2+^ ions as a cofactor for DHPS activity.^48,49^

**Figure 5:**
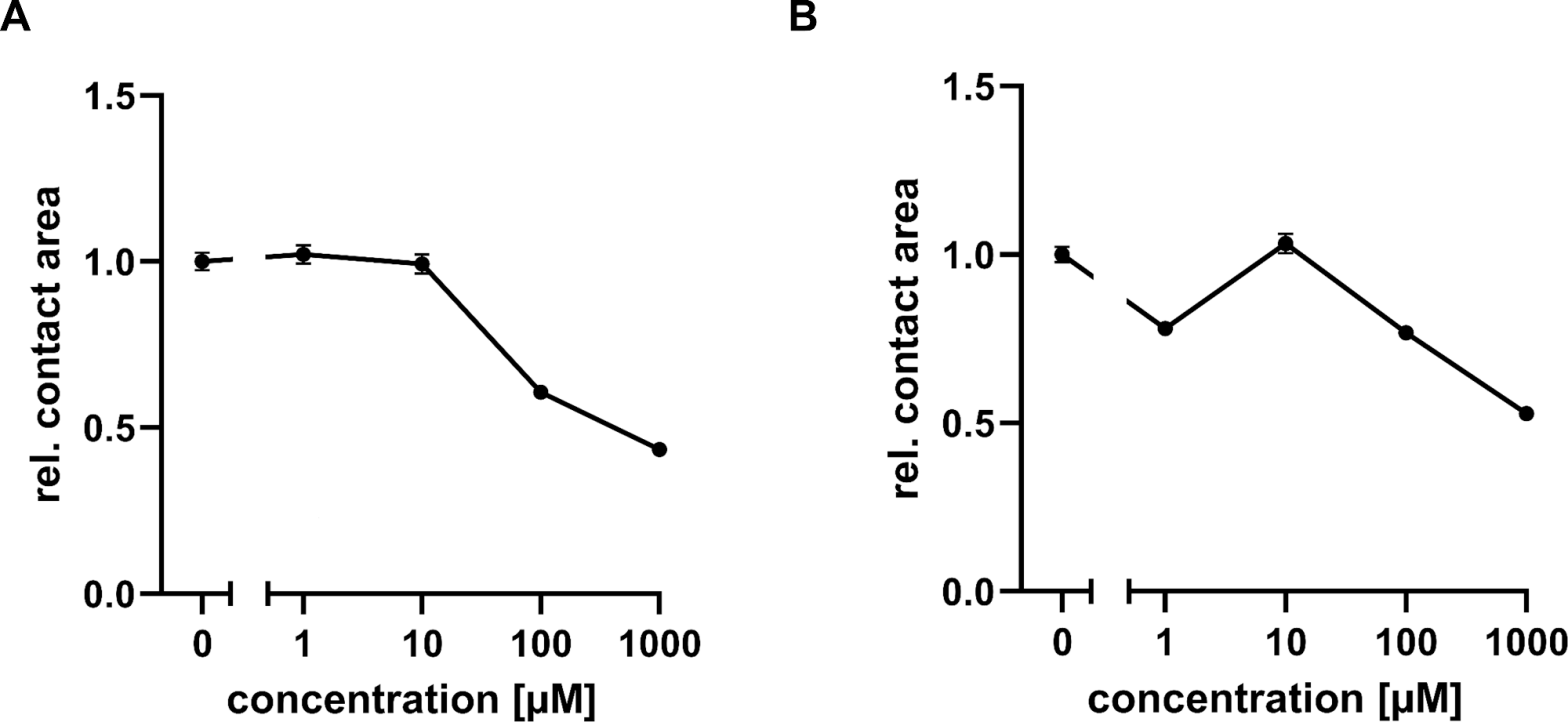
Biosensing SCP assay for the detection of sulfamethoxazole (SMX). Detection of SMX on a biochip surface with DHPS coupling via PEMA (A) and POMA (B) coating. SMX was detected in a concentration range between 0 μM and 1 mM and determined by the change of SCP contact area diameter relative to the negative control with no free SMX in assay buffer. Relative SCP contact area diameter is shown as mean ± SEM of at least 320 SCP (for PEMA) and 270 SCP (for POMA).

To demonstrate the functionality of the sulfonamide biosensing assay, we spiked the assay buffer with different concentrations of SMX as free analyte ranging from 0 to 1 mM. Resulting SCP contact areas were detected via RICM. For DHPS immobilization on PEMA coated surfaces, we observed a concentration dependent interaction between the SCP and the immobilized enzyme. SCP contact area diameter decreased with an increasing SMX concentration (Figure 5A). A clear difference in contact area diameter was detected for concentrations above 10 μM free SMX, with a diameter decrease of over 50% at the highest measured concentration of 1 mM SMX. This result illustrates that SMX can be successfully detected in our biosensing assay setup.

In another set of experiments, we investigated the interaction between functionalized SCP and the DHPS alternatively coupled to the chip surface using the more hydrophobic POMA coating. Our measurements showed again a concentration dependent decrease in the SCP contact area diameter above 10 μM SMX, while data considerably fluctuated in the concentration range below 10 μM (Figure 5B). While both coupling schemes allow for a nonoriented covalent DHPS immobilization, the more hydrophobic POMA surfaces led to a higher protein density on the biochip surface compared to PEMA (Figure 4B). While a high protein density might be beneficial for a stronger interaction with the SMXder functionalized SCP probes at first sight, the high protein density can also lead to steric hindrance of neighboring DHPS enzymes and disturbance of the binding sites. Also, hydrophobic interactions of proteins with the biochip surface can result in conformational changes of the protein impeding analyte binding by disrupting the enzyme binding pocket, in accordance to previous studies.^24,25,39^ These possible impacts of the hydrophobic polymer coating of the biochip on DHPS performance can be reasons for the observed data fluctuation, suggesting more reliable usage of PEMA coatings for DHPS coupling to glass biochips.

**Figure 6:**
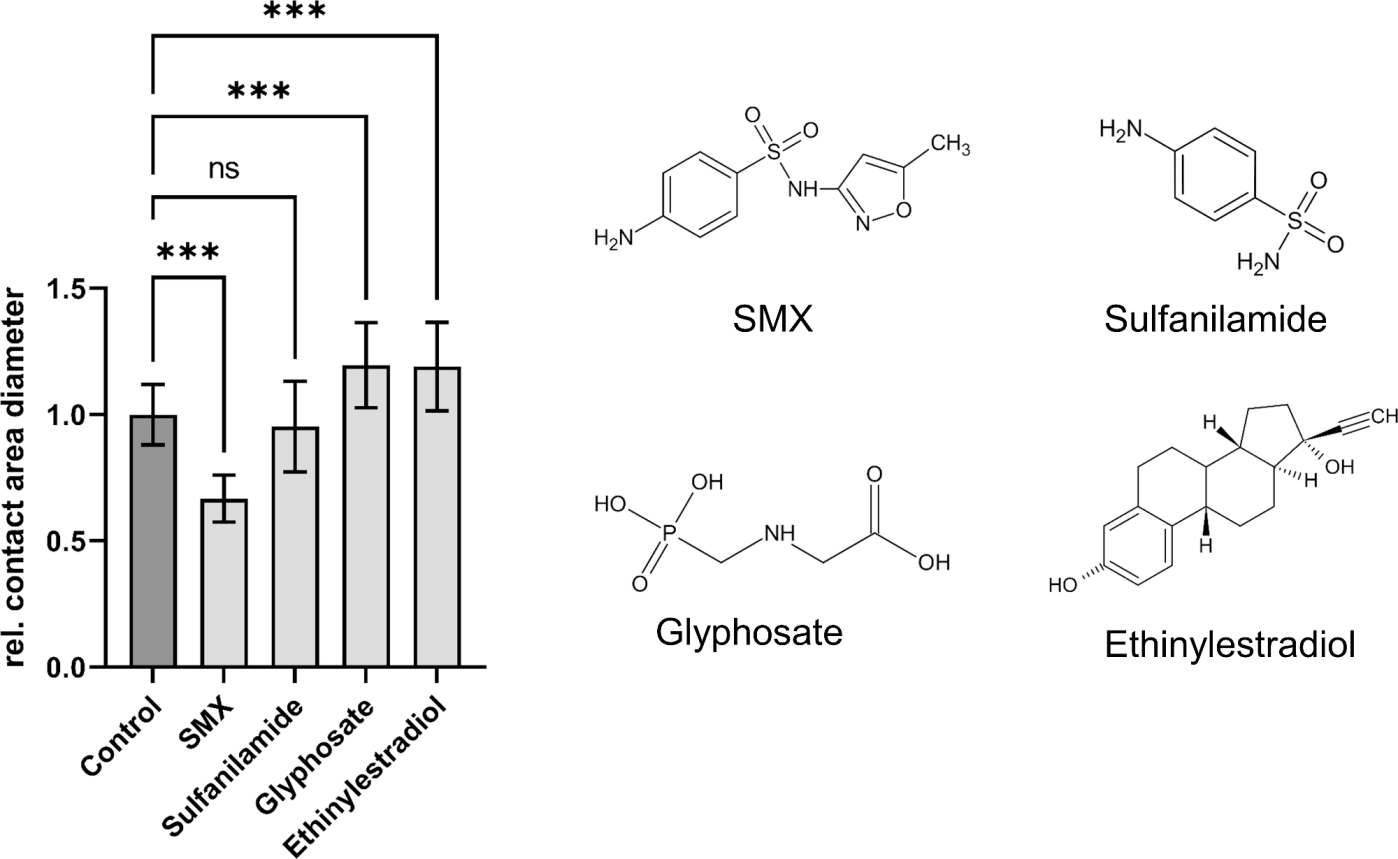
Selectivity of the biosensing SCP assay. Detection of SMX, sulfanilamide, glyphosate and ethinylestradiol on a biochip surface with DHPS immobilized on PEMA-coating and SMXder functionalized SCP. All analytes were applied at a concentration of 100 μM. No analytes were added in the control condition. Selectivity was determined by the change in contact area diameter relative to the control condition. Relative contact area diameter is presented as mean ± SD of at least 130 SCPs.

After demonstrating the successful detection of SMX with DHPS coupled via PEMA on the biochip, we used this setup and investigated whether other low molecular weight analytes can also lead to a decrease in contact area size and thus have an interfering effect on the biosensing assay performance. Since pesticides and hormones have a strong presence in the environment we chose glyphosate and ethinylestradiol as representatives.^50,51^ Additionally, by using sulfanilamide, we tested if other sulfonamide antibiotics besides SMX could be detected with our biosensing assay as well. We used 100 μM of either SMX, sulfanilamide, glyphosate or ethinylestradiol and evaluated the change in contact area diameter relative to the control condition that only contained SXMder functionalized SCPs interacting with DHPS on the biochip (Figure 6**)**.

As expected and demonstrated in the proof-of-concept experiments, the SCP contact area decreased upon addition of SMX to the assay (Figure 6). In contrast to SMX, we could not detect a significant decrease of contact area upon adding sulfanilamide. Considering the DHPS binding affinity for different sulfonamide antibiotics, the dissociation constant (*K*_d_) for SMX is 9.5 μM, whereas the *K*_d_ for sulfanilamide is 31 μM.^23^ Thus, the binding of sulfanilamide is too weak for detection in the current setup, also indicating the high specificity of our new assay. However, the advantage of using hydrogel microparticles for the SCP biosensing assay is that the detection sensitivity strongly relies on SCP parameters including particle elasticity, analyte’ competitor coupling, and buffer conditions. Decreasing the SCP’s elastic modulus leads to a larger contact area and a stronger SCP deformation providing a higher sensitivity. Elasticity of SCP is known to be adjusted by decreasing the PEG concentration and cross-linking density during SCP synthesis.^29^

Then, testing for interfering substances, for both glyphosate and ethinylestradiol, we detected some increase of the contact area compared to the control, indicating that the binding between the SCPs and the biochip surface is slightly strengthened in the presence of these molecules. While this observed increase in SCP-biochip interaction might be attributed to osmotic effects in the currently nonoptimized buffer solution and salt bridges,^52^ it clearly indicates that these molecules do not competitively interfere with SMX-DHPS binding. It needs to be considered, too, that we used a quite high analyte concentration of 100 μM, which is much higher than usually occurs for pesticides and pharmaceutics in environmental samples. Overall, these results confirm the specific detection of SMX by the decrease of the contact area diameter in the presence of SMX which was not observed for the control analytes sulfanilamide, glyphosate, and ethinylestradiol.

Taken together, we showed the feasibility of using the SCP based biosensing assay for the detection of sulfonamide antibiotics using SMX as a representative molecule being one of the most abundant sulfonamide antibiotics found in the environment.^53^ Our proof-of-concept study demonstrates the detection of SMX above a concentration of 10 μM in aqueous samples. The straightforward recombinant protein production of DHPS and immobilization on the biochip surface by reactive polymer coatings allow its application in a cheap and fast point-of-use biosensor setup. Furthermore, we could show that our current biosensing assay specifically detects SMX. We are confident, that it has the potential for an extension to detect other sulfonamide antibiotics with a higher *K*_d_ than SMX by using SCPs with a lower elastic modulus.

Although there are still no regulations regarding a concentration threshold for sulfonamide antibiotics in the environment in Germany, the European Commission increases efforts to monitor SMX which is emphasized by its inclusion in the EU Watch List for surface water contaminants in 2020.^54,55^ Published concentrations of SMX can reach up to 100 μg/l (ca. 0.4 μM) in the environment.^56,57^ In our proof-of-concept study, we did not detect concentration-dependent changes in the SCP contact areas below a concentration of 10 μM SMX up to now. However, the modularity of our SCP based biosensing platform provides many options to further increase the assays’ sensitivity as shown previously.^18,21^ Crucial parameters having strong effects on the assay sensitivity are the chemistry and length of the linker for analyte coupling to the SCP as well as SCP elastic modulus. The extension of the SCP assay toward very low detection limits was already shown for the detection of glyphosate, which even allowed a picomolar sensitivity.^16^ Moreover, the immobilization strategy of the enzyme and therefore the availability of its binding site on the biochip surface as well as used buffer conditions for tuning the specific binding of SMXder and DHPS are relevant parameters in adjusting the sensitivity and will be addressed in forthcoming works.

## 5 Conclusion

In this work, we report the successful detection of sulfonamide antibiotics in aqueous solution using a specific biomimetic binding principle with elastic hydrogel microparticles, so-called SCP, functionalized with an SMX derivative. Successful immobilization of recombinantly produced enzyme DHPS as a recognition element was demonstrated on either PEMA- or POMA-coated biochips in an active conformation. This fact nicely indicates that the straightforward nonoriented covalent coupling of the natural target of SMX, the enzyme DHPS, can be implemented in a competitive SCP assay format. Furthermore, the new functional coupling of SMX via an SMXder to monodisperse SCP improves the applicability in a fast and easy readout format for the SCP assay. With SMX as one of the most abundant sulfonamide antibiotics, we proved the general working principle by specifically detecting SMX in the micromolar range.

However, sulfonamide antibiotics occur in concentrations below our current detection assay performance in the environment.^56,57^ Therefore, following work will need to focus on sensitivity improvements and optimization of the biosensing setup by using softer SCPs, different linker chemistries for SMXder coupling to SCP and different immobilization strategies for the recognition element. As the complex composition of environmental samples can have an influence on assay performance, buffer conditions and sample composition especially regarding interfering substances have to be studied, e.g., concerning pH, salt ions, heavy metal ions, endocrine disruptors, and herbicides being present in different concentrations ranges.^50,51,58^ This comprehensive evaluation of the robustness and sensitivity of the biosensor toward real-world samples and potentially necessary sample pretreatment require further investigations. Nevertheless, with the presented results, we built the basis to extend the SCP sensor platform providing the proof-of-concept for a cost-effective, easy-to-use, and fast detection setup to monitor sulfonamide antibiotics in the environment at point of use. This concept will allow lab-based high precision analytics to be supported with cheap and fast point-of-use monitoring options at sufficient sensitivity.

## Supporting Information Available

Biochemical analysis of DHPS enzyme with MALDI-ToF-MS mass spectrum and size exclusion chromatogram of purified DHPS enzyme; amino acid sequence, and nucleic acid sequences of generated DHPS enzyme (PDF)
