# Supplementary material for: Detection of Sulfonamide Antibiotics Using an Elastic Hydrogel Microparticles-Based Optical Biosensor
Source: ACS Appl Mater Interfaces. 2024 Sep 13;16(38):50202–11. doi: 10.1021/acsami.4c08010 (PMC11440465; doi:10.1021/acsami.4c08010)
Supplement: Supplementary file 1 — am4c08010_si_001.pdf [file am4c08010_si_001.pdf]

Supporting Information

Detection of Sulfonamide Antibiotics Using Elastic Hydrogel Microparticles Based Optical Biosensor

*Veronika Riedl [a], Lara Heiser [a], Matthias Portius [a], Jann Ole Schmidt [a], Tilo Pompe [a]**

[a] Institute of Biochemistry, Leipzig University, Johannisallee 21-23, 04103, Leipzig, Germany

* corresponding author: tilo.pompe@uni-leipzig.de

1. DHPS Protein Analysis

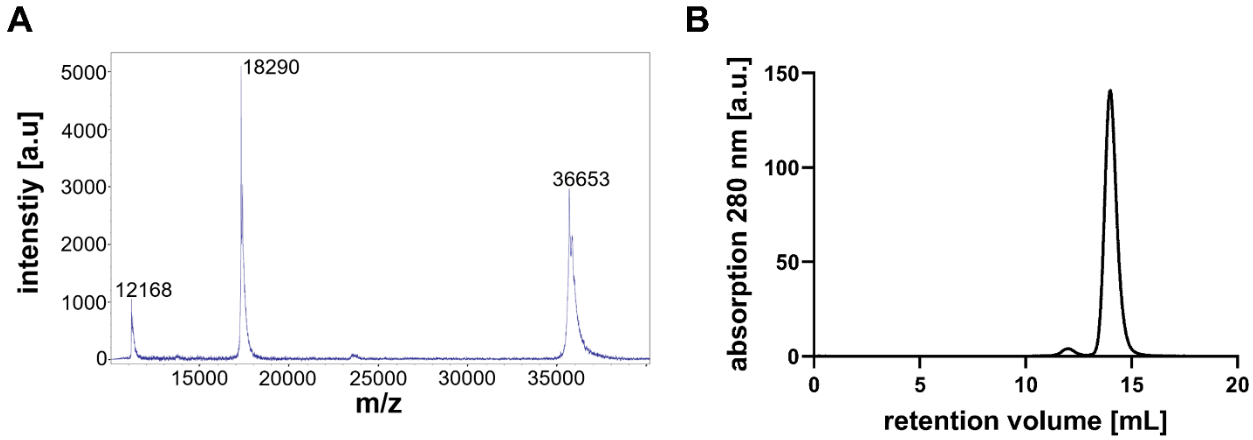

Figure S1: Biochemical analysis of DHPS after expression and purification in E. coli. **A**: Matrix-assisted laser desorption/ionization time-of-flight (MALDI-ToF) mass spectrum showing the peaks for the charged DHPS molecule ions and confirming the expected molecular weight of 36.9 kDa. **B:** Size exclusion chromatogram of DHPS purification.

2. His$_6$-DHPS Generated Fusion Protein Amino Acid Sequence

MGSSHHHHHHSSGLVPRGSHMASMSSKANHAKTVICGIINVTPDSFSDGGQFFALEQAL

QQARKLIAEGASMLDIGGESTRPGSSYVEIEEEIQRVVPVIKAIRKESDVLISIDTWKSQVA

EAALAAGADLVNDITGLMGDEKMPHVVAEARAQVVIMFNPVMARPQHPSSLIFPHFGF

GQAFTEEELADFETLPIEELMEAFFERALARAAEAGIAPENILLDPGIGFGLTKKENLLLL

RDLDKLHQKGYPIFLGVSRKRFVINILEENGFEVNPETELGFRNRDTASAHVTSIAARQG

VEVVRVHDVASHRMAVEIASAIRLADEAENLDLKQYK*

3. His$_6$-DHPS Generated Fusion Protein Nucleic Acid Sequence

Atgggcagcagccatcatcatcatcatcacagcagcggcctggtgccgcgcggcagccatatggctagcatgtcaagtaaagccaatcat

gcaaagacagttatttgcggaattatcaatgtaaccccagactccttttcggacggtggtcaatttttgctcttgagcaggcactccagcaggc

tcgtaaattgatagcagaaggagccagtatgctcgatatcggcggagaatcgactcggccgggcagtagctatgttgagatagaagagga

aatccagcgtgttgttccagtgatcaaagcgattcgcaaggaaagtgatgtcctcatctctattgatacttggaagagccaagtagcagaggc

tgctttggctgctggtgccgatctagtcaatgatatcactggtcttatgggtgatgagaaaatgcctcatgtggtagctgaagcgagagcgca

agtggtcatcatgtttaatccagttatggcgcgacctcagcaccctagctcgctcatcttccctcattttggttttggtcaagcttttacagaggaa

gagttagctgactttgaaacattgccaatcgaagaattgatggaggctttctttgaacgagcactagcgagagcggcagaagctggtattgca

ccagaaaatatcctgttggatccaggaattggctttggtctgaccaagaaagaaaatctgcttcttttacgggacctggataaactacatcaga

agggctatccaatctttctcggagtgtcgcgcaagcgatttgtcatcaatatcctagaggagaatggttttgaagtcaatcctgagacagagct

tggtttccgcaatcgggacacggcttcggctcatgtaaccagtatcgctgcaagacagggtgtagaagtggtgcgcgtgcatgacgtagct

agtcacaggatggcagttgaaattgcctctgccattcgtctggctgatgaagcggaaaatttagatttaaaacaatataaataa*
